# Mould-Free Microneedles in a Single Step: 3D Printing with Photopolymer Resins for Transdermal Delivery

**DOI:** 10.3390/pharmaceutics17111498

**Published:** 2025-11-19

**Authors:** Rutuja N. Meshram, Dimitrios A. Lamprou

**Affiliations:** School of Pharmacy, Queens University Belfast, 97 Lisburn Road, Belfast BT9 7BL, UK; rmeshram01@qub.ac.uk

**Keywords:** 3D printing, digital light processing, dissolving microneedles, transdermal, drug delivery, photopolymerisation

## Abstract

**Background:** Digital light processing (DLP) 3D printing has emerged as a rapid alternative to labour-intensive micro-moulding for producing microneedle (MN) arrays, yet its use in biodegradable, dissolving MNs has been limited by proprietary, non-degradable resins. **Methods:** The current study proposed an innovative, biocompatible PEGDA–vinyl-pyrrolidone photo-resin with lithium phenyl(2,4,6-trimethylbenzoyl) phosphinate initiator, which systematically optimises its rheology and photo-reactivity for DLP printing. Resin formulations were evaluated through viscosity profiling, cure kinetics, FTIR, and ^1^H NMR, and MN arrays were printed using a desktop DLP platform and characterised by optical microscopy, mechanical testing, thermal analysis, and dissolution studies. **Results:** A 40% PEGDA up-to 100% VP blend with 0.4% initiator was identified as providing rapid photopolymerisation, low shrinkage and complete vinyl conversion. Using a desktop DLP platform, 6 × 6 MN patches were printed in a single step without moulds and analysed by optical and scanning electron microscopy. The printed MNs reproduced CAD dimensions with <3% deviation, achieving a height of 1.40 ± 0.02 mm and a base thickness of 1.00 ± 0.01 mm, and showed a tip radius consistent with sharp penetration. Compression testing measured an array force of 32 N, corresponding to ~0.9 N per needle, exceeding the 0.2 N threshold for skin insertion. FTIR and ^1^H NMR confirmed near-quantitative crosslinking, thermogravimetric and differential scanning calorimetry indicated stability at ambient conditions, and dissolution studies showed complete needle dissolution. **Conclusions:** An optimised PEGDA/VP resin yields geometrically precise, mechanically robust dissolving MNs in a single step, addressing the limitations of micro-moulding and paving the way for customisable, on-demand transdermal delivery of active molecules and biologics.

## 1. Introduction

The skin, the body’s largest organ, serves as a multi-layered protective barrier against chemical, microbial, and mechanical challenges [1]. Anatomically, it comprises the epidermis, dermis, and hypodermis [2], but the outermost ~10–25 μm stratum corneum (SC) is the principal barrier to exogenous molecules. The SC “brick-and-mortar” structure of corneocytes embedded in a lipid matrix, effectively limits permeation that only a few drug compounds (<500 Da, log P ~2–4, high potency) can cross intact skin unaided [3]. As a result, most hydrophilic drugs, macromolecules, and vaccines still rely on hypodermic needle injection, an invasive approach associated with pain, needle-stick injuries, cold-chain storage requirements [4], and poor patient compliance. Various strategies (e.g., chemical enhancers, iontophoresis, ultrasound via sonophoresis [5,6]) have been explored to expand transdermal delivery, but offer only modest enhancement and can pose safety issues, limiting widespread use. MN arrays provide a promising physical means to bypass the superficial barrier of the skin. These devices consist of microscopic projections (typically ~150 μm to 1.5 mm in length) [7] that pierce the SC to create transient microchannels for drug or vaccine delivery, while avoiding nerves and blood vessels to minimise pain and bleeding. Ref. [8] MNs come in several designs (solid, coated, hollow, hydrogel-forming, and dissolving), among which dissolving microneedles (DMNs) are particularly attractive because they are made from water-soluble, biocompatible polymers that dissolve or biodegrade in the skin, eliminating sharps waste and allowing controlled drug release. Notably, DMN patches have successfully delivered biologics (e.g., peptides, proteins, nucleic acids) that would be unstable in the gastrointestinal tract or unable to cross intact skin [9,10]. However, an effective DMN must satisfy two opposing requirements: (i) sufficient mechanical rigidity to penetrate the SC, and (ii) rapid polymer dissolution to release the drug load shortly after insertion. Achieving this trade-off depends critically on both the polymer formulation and the fabrication method.

Conventional fabrication of DMNs relies on micro-moulding: a master mould is replicated in an elastomer like polydimethylsiloxane (PDMS), and a liquid polymer solution is cast into these cavities, then degassed, cured, and demoulded to yield MN arrays [11]. While straightforward, moulding can introduce geometric imperfections, blunted tips, tapered sidewalls, and incomplete filling that diminish insertion efficiency and batch uniformity. Moreover, the multi-step moulding workflow hinders rapid design iteration and complicates scale-up. These limitations have driven interest in additive manufacturing (3D printing) as a direct-write alternative to produce custom MN arrays on demand without moulds [12]. It is transforming healthcare by enabling the rapid production of highly personalised MNs with precisely tailored dimensions and drug loadings [13], while thinking about sustainability, its efficient, layer-by-layer production model simultaneously reduces material waste and simplifies scale-up compared to traditional moulding [14]. Among the 3D-printing methods, digital light processing (DLP) has emerged as a promising route for MN fabrication [15]. DLP uses a digital micromirror device to project patterned light onto a vat of photocurable resin, polymerising each layer in a single flash exposure. This parallel curing approach, unlike point-by-point laser stereolithography (SLA), achieves sub-50 μm feature resolution, uniform exposure across the layer, and build times of only seconds per layer [16] also new system can achieve higher resolution than this. Bottom-up DLP configurations further minimise peel forces during layer separation, enabling high-aspect-ratio structures with sharp tips. Indeed, DLP-printed polymer lattices have demonstrated higher strength-to-density ratios than moulded counterparts, attributed to more complete monomer conversion and lower porosity [17]. A major challenge for DLP-printed MNs is the limited biocompatibility of standard resins. Most commercial photopolymers use non-degradable acrylate matrices and toxic photo-initiators, rendering them unsuitable for DMNs. To address this, researchers have developed inks using degradable hydrophilic monomers like poly (ethylene glycol) diacrylate (PEGDA) and N-vinyl-2-pyrrolidone (VP) combined with water-soluble photoinitiators (e.g., LAP) [18]. However, the work from Petrova et al. [18] primarily addressed drug loading and release, without systematically examining the rheological behaviour or cure kinetics of the resin, evaluating monomer conversion, assessing the mechanical performance of the MNs, or exploring multiple geometrical designs. In contrast, the present study comprehensively investigates these parameters to establish a deeper understanding of formulation–process–performance relationships in PEGDA/VP-based DMNs fabricated via DLP printing. Co-monomers such as VP can be added to lower resin viscosity and improve print fidelity [13]. Adjusting the PEGDA/VP ratio and photoinitiator loading allows tuning of viscosity, crosslink density, mechanical strength, and dissolution rate, though these parameters have yet to be systematically optimised for DMNs. Only isolated studies have so far attempted to fabricate DMNs directly via DLP printing [12,13,18], often using proprietary resin formulations and providing limited mechanical data. As a result, the relationships between resin composition, print fidelity and device performance remain poorly understood. Therefore, a comprehensive investigation is needed that (i) specifies the printable formulation window for degradable PEGDA/VP resins, (ii) correlates formulation and exposure parameters with MN geometry and mechanical performance, and (iii) benchmarks DLP-printed DMNs against conventional mould-cast analogues. Addressing these gaps will determine whether vat photopolymerisation can produce MN patches with the required precision, mechanical robustness, and fast dissolution for clinical applications. In the current study, these questions are tackled by formulating a range of biodegradable PEGDA/VP resin blends (PEGDA/VP with LAP photoinitiator) and evaluating their viscosity, photo-reactivity, and printability. Using these resins, designed DMN arrays (conical and pyramidal, different heights as well as base radius) and printed them on a desktop DLP system under optimised conditions to limit dimensional deviation of the computer-aided design (CAD). The printed MNs were comprehensively characterised using a range of microscopic, spectroscopic, and thermal analysis techniques. Real-time spectroscopic monitoring was employed to track the progression of photopolymerisation, while optical and scanning electron microscopy were utilised to characterise microneedle geometry and surface morphology. Rheological analysis was conducted to determine the appropriate shear-rate conditions for stable bottom-up printing, and axial compression testing was applied to assess mechanical strength relative to reported skin-insertion thresholds. Dissolution studies in physiological buffer were further undertaken to examine crosslink density and matrix stability. Collectively, these methodologies were selected to provide a comprehensive evaluation of the DLP process and to establish its potential advantages over traditional mould-based fabrication for producing uniform, mechanically reliable MNs. By elucidating these formulation-process, performance relationships, this study establishes DLP 3D printing as a robust single-step platform for on-demand production of DMN patches for transdermal delivery.

## 2. Materials and Methods

### 2.1. Materials

N-vinyl-2-pyrrolidone (VP, ≥98%), polyethylene glycol diacrylate (PEGDA) with a molecular weight of 700 g/mol, and lithium phenyl-2,4,6-trimethylbenzoylphosphinate (LAP, ≥95%) were obtained from Sigma Aldrich, part of Merck KGaA (Darmstadt, Germany). Phosphate-buffered saline (PBS) tablets, at pH 7.4, were also included. Deuterated dimethyl sulfoxide-D6 + 0.03% 1,3,5 trioxane (DMSO-d_6_; ≥98%; Sigma-Aldrich, St. Louis, MO, USA), purchased from VWR International (Lutterworth, UK), was used as supplied. Commercial resin for DLP printer PlasCLEAR^®^ 3DP (Poly (methyl methacrylate) PMMAs) Polymers were purchased from iMakr (London, UK). Isopropyl alcohol (IPA) was purchased from Sigma Aldrich (St. Louis, MO, USA). Methylene blue (≥97%) was obtained from Merck KGaA (Darmstadt, Germany). The water used in the experiments was deionised, distilled, and subsequently filtered through a Millipore Q purification system.

### 2.2. Optimisation of CAD Design and DLP Printing Parameters

**Figure 1:**
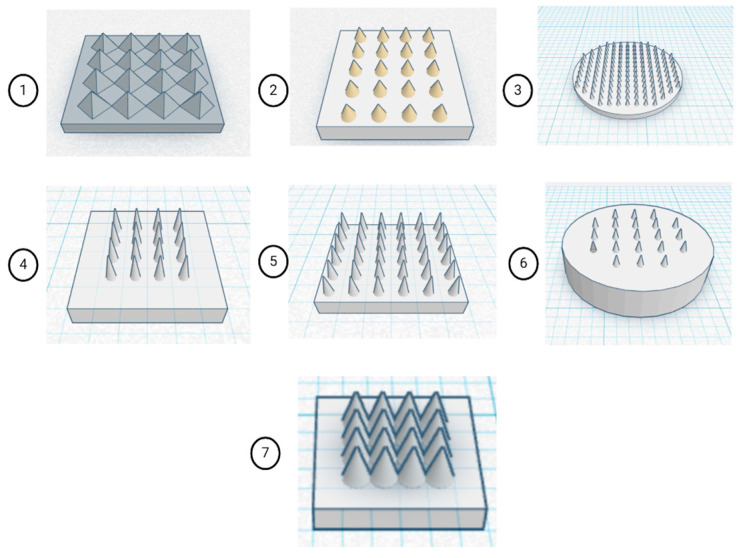
CAD models of MN array geometries created in Tinkercad (Autodesk, USA) showing systematic variation in patch dimensions, needle number, and shape to evaluate structural and functional performance. In which (1) 14 × 14 × 1 mm, 16 pyramidal MNs; (2) 12 × 12 × 1.5 mm, 20 conical MNs; (3) 12 × 12 × 0.5 mm, 50 conical MNs (circular); (4) 10 × 10 × 1 mm, 16 conical MNs; (5) 10 × 10 × 1 mm, 36 conical MNs; (6) 8 × 8 × 2 mm, 21 conical MNs (circular); (7) 6 × 6 × 1 mm, 16 conical MNs.

Multiple MN geometries were constructed using Tinkercad (Autodesk, San Francisco, CA, USA), an open-access CAD platform. The selection of multiple MN geometries was guided by the need to evaluate how structural parameters affect functional performance systematically. Variations in needle height and base radius were introduced to balance mechanical strength and penetration depth, while tip sharpness was optimised to facilitate efficient skin insertion with minimal tissue damage. Differences in patch configuration and needle density were designed to study their influence on dissolution kinetics across the array. These models comprised both pyramidal and conical MN architectures, arranged within circular or rectangular patch configurations, as depicted in Figure 1. Both geometries were selected as they represent the most reported morphologies for DMNs, offering high tip sharpness, uniform stress distribution, and reliable insertion into the skin while maintaining structural stability during fabrication and penetration. Design parameters such as patch dimensions, number of MNs per array, individual needle height, and base radius were systematically varied, as summarised in Table 1. Asiga Composer V1.2.11. slicer used for slicing of Standard Tessellation Language (.STL) files. Preliminary fabrication was performed using a commercial transparent resin (PlasCLEAR^®^) to validate print accuracy and assess geometric fidelity. Based on morphological evaluation, a single optimised design was selected that demonstrated superior tip sharpness, uniform array distribution, and structural integrity. Subsequent optimisation of DLP printing parameters, including light intensity, slice thickness, and exposure time, was conducted to achieve consistent layer curing and minimise dimensional deviation between the printed structures and the CAD design. This iterative approach ensured the production of high-resolution MN arrays with reproducible geometry suitable for further mechanical and functional evaluation.

### 2.3. Ink Preparation by Screening of Polymers

Photoinitiator LAP was accurately weighed and incorporated into PEGDA via vortex mixing until a visually homogeneous mixture was obtained. Subsequently, VP was added, and mixing continued until the solution became fully optically clear. A series of resin formulations featuring in *w*/*w*% PEGDA/VP weight ratios of 50%:50% (1:1), 10%:90% (1:9), 40%:60% (2:3), and 30%:70% (3:7) were prepared. A range of PEGDA/VP weight ratios was selected to optimise the resin’s rheological behaviour and crosslinking potential during MN fabrication. Increasing the VP fraction raises resin viscosity, which enhances vertical resolution and facilitates the printing of high-aspect-ratio MNs. This ratio variation enabled the identification of compositions compatible with bottom-up DLP printing, ensuring layer stability and accurate geometry reproduction. LAP concentration was varied between 0.1% and 0.5% *w*/*w* to determine the minimum effective level for complete photopolymerisation. Each was stored in amber colour vials for further use. Viscosity behaviour and flow dynamics were characterised via shear-rate sweeps to ensure compatibility with bottom-up recoating in the DLP vat. Photopolymerisation efficiency was evaluated using real-time Fourier-transform infrared (FTIR) and Nuclear Magnetic Resonance (^1^H NMR) by monitoring the decrease in acrylate/vinyl bands. An ideal resin was identified based on its viscosity, rapid photopolymerisation, precise print fidelity, and stable polymer network. The optimised formulation exhibiting these characteristics is detailed in Section 3. 

### 2.4. Rheological Characterisation of the Inks

The rheological behaviour of the formulated resin was evaluated to determine its suitability for DLP 3D printing. Resin viscosity plays a crucial role in ensuring uniform recoating and preventing printing artefacts associated with excessive flow resistance. Measurements were performed using a rotational rheometer HAAKE™ MARS™ Rheometer (Thermo Fisher Scientific, Loughborough, UK) equipped with a parallel plate geometry (35 mm diameter) using an already developed protocol by Pitzanti et al. [19]. The shear rate was varied from 0 to 100 s^−1^ under controlled conditions at 25 °C. Each formulation was analysed in triplicate to ensure reproducibility. The apparent viscosity was recorded as a function of shear rate, and the steady-state viscosity was determined once a plateau region was reached, representing the equilibrium flow behaviour of the resin.

### 2.5. Fabrication of MN Arrays via DLP 3D Printing

MN arrays were fabricated DLP 3D printing technique. The photopolymerisable resin formulations were poured into the resin vat of an inverted DLP printer (Asiga Max X27, Asiga^®^, Sydney, NSW, Australia). The corresponding CAD models of MN arrays were subsequently processed in Asiga Composer software (version 1.2.11) for slicing and build preparation. Printing parameters, including layer thickness, exposure time per layer, and light intensity, were optimised to ensure complete crosslinking and high-fidelity replication of the CAD geometry.

Following fabrication, printed MN arrays were carefully rinsed with isopropyl alcohol to remove residual uncured resin and then subjected to post-curing for 5 min in an Asiga Flash UV chamber to reinforce polymer network stability. The printed arrays were evaluated for structural integrity using both optical microscopy and scanning electron microscopy (SEM) to verify tip sharpness, dimensional uniformity, and absence of surface defects. Each batch was assessed for average patch mass, overall dimensions, and MN height. Only arrays conforming to the intended design specifications were selected for subsequent physicochemical and mechanical characterisation.

### 2.6. Fourier-Transform Infrared Spectroscopic Analysis

The chemical interactions and structural evolution of the photopolymerised resin were investigated using attenuated total reflectance, Fourier transform infrared (ATR-FTIR) spectroscopy. The analysis aimed to elucidate potential chemical bonding or physical interactions occurring during the photopolymerisation of PEGDA and VP. A physical mixture of PEGDA and VP was prepared by vortex mixing until a homogeneous dispersion was achieved. ATR-FTIR spectra of the individual polymers, uncured resin, and printed MN arrays were recorded using a Nicolet™ iS50 FT-IR spectrometer (Thermo Scientific™, Paisley, UK). Spectra were acquired in the wavenumber range of 4000–400 cm^−1^ with a spectral resolution of 4 cm^−1^, averaging 64 scans per sample after background correction. Each sample was analysed in triplicate to ensure reproducibility. Characteristic absorption bands were examined to identify functional group transformations indicative of photopolymer crosslinking and resin curing behaviour.

### 2.7. Proton Nuclear Magnetic Resonance Detection of Residual Monomers

Residual monomer content in the photopolymerised MN arrays was analysed using proton nuclear magnetic resonance (^1^H NMR) spectroscopy to confirm the extent of polymerisation. The printed MN arrays were finely ground using a mortar and pestle, and 10 mg of the resulting powder was dispersed in 1 mL of DMSO-d_6_ containing 0.03% TMS as an internal reference standard. The mixture was maintained at room temperature for 24 h to allow the extraction of any unreacted monomers, following the protocol described by [13]. After incubation, the samples were centrifuged, and 500 µL of the clear supernatant was collected for analysis. For comparison, 10 mg of the unpolymerized PEGDA/VP resin was also dissolved in 1 mL of DMSO-d_6_ + 0.03% TMS and analysed immediately under identical conditions. The ^1^H NMR spectra were acquired using a Bruker NMR spectrometer (Bruker BioSpin GmbH, Rheinstetten, Germany), and data were processed using Bruker TopSpin software 4.5.0. The disappearance or reduction in characteristic vinyl proton signals was interpreted as evidence of double-bond consumption during photopolymerization. All analyses were performed in triplicate to ensure reliability and reproducibility.

### 2.8. Thermal Analysis

#### 2.8.1. Thermogravimetric Analysis

Thermogravimetric analysis (TGA) was employed to evaluate the thermal stability of the individual polymer components and to confirm polymerisation in the printed MN arrays. Although printing was performed at ambient room temperature, well below the degradation thresholds of the materials, the thermal decomposition profiles provided useful insight into the formation and stability of the crosslinked polymer network. TGA measurements were conducted using a Q50 thermogravimetric analyser (TA Instruments, New Castle, DE, USA). Approximately 5–10 mg of each sample was placed in an open aluminium pan and heated from 25 to 500 °C at a constant rate of 10 °C min^−1^ under a nitrogen atmosphere. The weight loss of each sample was continuously recorded as a function of temperature. Thermograms were processed and analysed using Universal Analysis software (version 5.5.24) to determine onset degradation temperatures (T_onset_) and weight-loss profiles. All analyses were performed in triplicate to ensure reproducibility and accuracy.

#### 2.8.2. Differential Scanning Calorimetry

Differential scanning calorimetry (DSC) was conducted to evaluate the thermotropic transitions and phase behaviour of the fabricated MN arrays. The analysis provided insights into the thermal stability, melting behaviour, and potential polymer, polymer interactions within the crosslinked matrix. Thermal measurements were performed using a DSC 214 Polyma instrument equipped with an autosampler (NETZSCH GmbH & Co. Holding KG, Selb, Germany). Approximately 7–8 mg of each sample was accurately weighed and hermetically sealed in aluminium pans, with an empty aluminium pan used as a reference. The samples were heated from −10 °C to 400 °C at a constant rate of 20 °C min^−1^ under a nitrogen purge of 40 mL min^−1^ to prevent oxidative degradation. Each measurement was carried out in triplicate to ensure reproducibility. Thermograms were processed using the NETZSCH Proteus analysis software (NETZSCH-Gerätebau GmbH, Selb, Germany, version 9.7) to determine characteristic transition temperatures, including the glass transition (Tg) and melting temperature (Tm). The melting point of each sample was identified from the peak of the corresponding endothermic transition in the thermogram.

### 2.9. Morphological and Physical Characterisation of MN Arrays

The morphological and physical characteristics of the fabricated MN arrays were analysed to assess geometric fidelity, surface uniformity, and reproducibility of the printing process. Dimensional parameters, including MN height, base width, and overall patch thickness, were measured using a stereomicroscope (EZ4W, Leica Microsystems, Wetzlar, Germany). The obtained dimensions were compared against the corresponding CAD models to evaluate dimensional accuracy and potential print deviations.

Similarly, surface morphology and microstructural features were examined using a dual-beam focused ion beam scanning electron microscope (FIB-SEM; TESCAN Lyra3, Brno, Czech Republic). Samples were mounted on aluminium stubs using carbon adhesive tape to enhance surface conductivity before imaging. The average patch mass was determined using an analytical balance (Thermo Fisher Scientific, Loughborough, UK). All analyses were performed using three independent samples (*n* = 3) to confirm the consistency of the experimental results.

### 2.10. Mechanical and Insertion Performance of MNs

#### 2.10.1. Parafilm^®^-Based Insertion Analysis

The insertion capability of the MN arrays was assessed using a multilayer Parafilm^®^ (Bemis Company Inc., Neenah, WI, USA) model, which is widely employed as a skin simulant due to its comparable mechanical resistance to human stratum corneum. Ten layers of Parafilm^®^ (each 10 × 10 cm) were stacked to achieve a total thickness of approximately 1 mm. Insertion testing was performed using a Texture Analyser (TA.XTPlus, Stable Micro Systems, Surrey, UK) equipped with a cylindrical probe. The MN arrays were attached to the probe and inserted into the Parafilm^®^ stack at a constant speed of 1.19 mm s^−1^ under a compression force of 32 N, which was maintained for 30 s, following the method described by [18]. After insertion, the Parafilm^®^ layers were carefully separated, and the number of perforations in each layer was quantified using an optical microscope. The percentage of MNs successfully inserted was calculated according to Equation (1): All measurements were performed in triplicate (*n* = 3) to ensure reproducibility and statistical reliability.

(1) $$\mathrm{Insertion}\;\mathrm{Efficiency}\;(\%)=\frac{\mathrm{Number}\;\mathrm{of}\;\mathrm{holes}\;\mathrm{created}}{\mathrm{Total}\;\mathrm{number}\;\mathrm{of}\;\mathrm{MNs}\;\mathrm{on}\;\mathrm{the}\;\mathrm{patch}}\times 100$$

Additionally, Thumb press insertion was also tested on the 10-fold Parafilm^®^, where the MN patch was pressed against the Parafilm^®^ for 30 s. The created holes were observed along with the pre- and post-insertion heights of the MNs.

#### 2.10.2. Ex Vivo Insertion on Porcine Skin and Chicken Breast Models

Ex vivo models were employed to simulate realistic soft-tissue and cutaneous conditions for evaluating the insertion capability of MN arrays. Neonatal porcine skin, selected for its close histological and mechanical resemblance to human skin, and chicken breast (pectoralis major), chosen as a homogeneous soft-tissue model to assess insertion depth and deformation resistance, were used as representative biological substrates. Fresh chicken breast tissue was procured from a local supplier on the day of testing. Neonatal porcine skin was obtained from a licensed local abattoir in accordance with institutional and regional ethical regulations. Upon receipt, the skin was rinsed with distilled water, stored at −80 °C, and thawed to room temperature immediately before testing. For sample preparation, tissues were mounted on clean Petri dishes; hair was carefully removed from the skin surface using a disposable shaving blade. The samples were blotted dry and secured at the edges with laboratory tape to minimise lateral displacement during insertion. MN patches were applied manually using gentle, uniform thumb pressure for 30 s and subsequently removed. The tissue surface was imaged before and after MN application using an optical microscope under identical illumination and magnification settings. Image analysis was performed to quantify the insertion efficiency, defined as the visible microchannels. Each condition was evaluated using five independent MN arrays (*n* = 5).

### 2.11. Dissolution Behaviour, Swelling Dynamics, and Degradation Analysis of MNs

The dissolution, swelling, and degradation characteristics of the MN arrays were investigated to assess their physicochemical performance under physiologically relevant conditions. These studies were conducted to ensure optimal polymer hydration and predictable structural disintegration within the skin microenvironment.

#### 2.11.1. Dissolution Behaviour

The dissolution capacity of the MNs was evaluated in both deionised water and phosphate-buffered saline (PBS, pH 7.4), the latter selected to mimic the physiological pH range of the skin surface and interstitial fluids. Individual MNs were carefully excised from the patch using a sterile surgical blade and immersed in 10 mL of dissolution medium maintained under constant agitation at 32 ± 0.5 °C to mimic the natural skin environment, which is one of the criteria for the study of dermal formulation in vitro assessment [20]. Visual observations were recorded at predetermined intervals until complete dissolution occurred. Each experiment was performed in triplicate (*n* = 3).

#### 2.11.2. Swelling Study

The swelling behaviour of the polymeric MN matrices was assessed to understand their hydration kinetics and their correlation with drug release performance. Pre-weighed MN patches were immersed in 10 mL of PBS (pH 7.4) at 32 ± 0.5 °C. At defined time intervals (0.5, 1, 2, 4, 6, 8, 24, 48, and 72 h), the patches were removed, gently blotted to remove surface moisture, weighed, and re-immersed in the same medium. The percentage of fluid uptake (%FU) was calculated using Equation (2): where W_0_ and Wₜ represent the initial and time-dependent swollen weights of the MN patch, respectively. All experiments were conducted in triplicate (*n* = 3).

(2) $$\%\;\mathrm{Fluid}\;\mathrm{Uptake}=\frac{W_{t}-W_{0}}{W_{0}}\times 100$$

#### 2.11.3. Degradation Analysis

The degradation behaviour of the MN patches was examined to determine their structural integrity and dissolution kinetics over time. Pre-weighed patches were immersed in PBS (pH 7.4) and incubated at 32 ± 0.5 °C for predetermined time intervals (0.5, 1, 2, 4, 6, 8, 24, 48, and 72 h). At each interval, the patches were retrieved, blotted, dried, and re-weighed. The percentage degradation (%D) was calculated using Equation (3): where W_0_ and Wₜ represent the initial and time-dependent degradation weights of the MN patch, respectively. All degradation experiments were performed in triplicate (*n* = 3) to ensure reproducibility.

(3) $$\%\;\mathrm{Degradation}=\frac{W_{0}-W_{t}}{W_{0}}\times 100$$

### 2.12. Surface Wettability Assessment by Contact Angle Goniometry

The surface wettability of the fabricated MN patches was evaluated using contact angle goniometry (CAG) to assess hydrophilicity, which influences polymer-water interactions and dissolution behaviour. Measurements were conducted using a Biolin Theta Tensiometer (Biolin Scientific, Manchester, UK) at ambient room temperature. Before each measurement, the sample stage was levelled using the magnetic ball calibration method to ensure precise droplet positioning. A 5 µL droplet of deionised water was gently deposited onto the flat surface of the MN patch (excluding the needle region) using an automated micro syringe. The dynamic contact angle was monitored for 60 s after droplet deposition, and the mean value over this period was recorded using One-Attention software (version 1.8, Biolin Scientific). All measurements were performed on three independent MN samples (*n* = 3), and results were expressed as mean ± SD.

### 2.13. Statistical Analysis

All experimental data were expressed as mean ± SD based on a minimum of three independent replicates (*n* = 3) for each condition. Statistical significance was assessed using one-way analysis of variance (ANOVA) to compare group means, with a probability value of *p* ≤ 0.05 considered statistically significant. Data analysis and graphical representations were performed using GraphPad Prism software (version 6.0, GraphPad Software, Inc., San Diego, CA, USA)

## 3. Results and Discussion

### 3.1. Optimisation of CAD Design and DLP Printing Parameters

The MN array design and DLP printing parameters were essential to achieve high-fidelity structures that accurately reproduced the intended CAD geometry. In DLP-based 3D printing, the lateral resolution is determined by the projected pixel pitch of the optical system, while the axial resolution depends on the interplay between layer thickness and the resin cure depth [21]. In the absence of a UV absorber, excessive light penetration can extend polymerisation beyond the target voxel boundaries, resulting in over-curing, edge distortion, and tip blunting [22].

**Figure 2:**
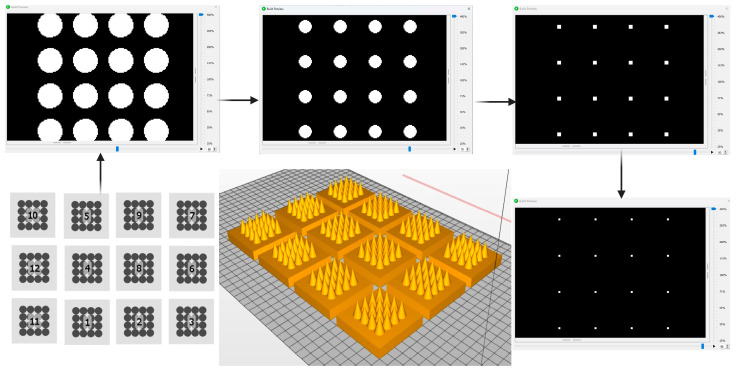
Illustration of the pixel-aware slicing and design optimisation process in digital DLP 3D printing of MNs. The process starts with a library of optimisation patterns (**bottom**-**left**) to generate a 3D model (**bottom**-**centre**). During slicing, sequential 2D cross-sections are produced: the base layer with the largest cross-section (**top-left**), intermediate layers showing gradual reduction in feature size as the needle tapers (**top-centre**), near-tip layers with small pixel clusters (**top-right**), and the final tip layer (**bottom-right**). These slices guide layer-by-layer fabrication of the MN array (6 × 6 mm patch, 16 needles) using pixel-aware DLP printing.

To mitigate these effects, a pixel-aware slicing strategy was implemented. The slicing pattern was aligned to the projector’s native pixel grid to preserve geometric fidelity, particularly in regions of high curvature such as the needle tips. A binary (on/off) exposure mode was employed near the tip region to prevent partial-cure edge voxels, while layer thickness was selectively adjusted across the build. Thicker layers were used in the base region to reduce the overall number of layers and peel cycles, improving build stability and minimising print time. In contrast, thinner layers were applied toward the needle tips to accurately capture curvature and maintain the designed base radius. As shown in Figure 2, the printed arrays exhibited precise reproduction of the CAD geometry, demonstrating uniform tip formation and consistent base dimensions without the appearance of pixel-induced artefacts. Morphological evaluation confirmed the high dimensional fidelity between digital and printed structures, indicating successful control of photopolymerisation spread and voxel overlap.

A systematic exploration of CAD configurations was conducted using the commercial PlasCLEAR^®^ resin to identify an optimal MN architecture that balances manufacturability and selection of an optimised design for a polymeric MN array. Seven distinct MN array geometries (Designs 1–7) were modelled using Tinkercad and subsequently visualised under a digital light microscope to evaluate dimensional accuracy and geometric fidelity. The tested designs varied in patch dimensions, microneedle count, height, and base radius. The pyramidal geometry was included as a reference design, whereas conical MNs were used for subsequent optimisation due to improved print fidelity and mechanical performance. Comparative evaluation revealed that the 6 × 6 × 1 mm patch containing 16 microneedles, each with a 1.5 mm height and 0.6 mm base radius (design 7), produced the most desirable morphological features. This configuration demonstrated sharp tip formation, structural uniformity, and mechanical integrity during post-processing, outperforming larger or denser array configurations that exhibited partial merging or tip deformation, since it was selected as an ideal design for the fabrication of polymeric MN array.

Overall, the integration of a pixel-calibrated slicing approach with geometrically optimised CAD design yielded MN arrays of high precision and reproducibility. These findings underscore the importance of coupling digital design refinement with exposure control in DLP-3DP to ensure accurate voxel confinement and reliable replication of micro-scale biomedical geometries.

### 3.2. Ink Preparation by Screening of Polymers

The resin constituents possess favourable safety profiles, such as PEGDA is a derivative of polyethene glycol and is noted for flexibility, non-toxicity, non-immunogenicity and hydrophilicity [23]. Polyvinylpyrrolidone (PVP), the polymerised form of VP, is recognised by the U.S. FDA as “generally recognised as safe” and is approved for oral, topical and injectable formulations. The formulation of a photopolymerisable ink represents a critical determinant of print fidelity, mechanical robustness, and downstream performance of DMNs. In this study, resin systems comprising PEGDA, VP, and LAP were systematically investigated to delineate a compositional window that supports both printability and mechanical functionality. The ratio of PEGDA to VP exhibited a pronounced influence on the curing kinetics and structural behaviour of the printed arrays. PEGDA-rich formulations produced softer and more compliant structures due to the flexible polyether backbone and lower crosslink density, whereas VP-rich blends yielded brittle arrays with reduced fracture resistance. This compositional sensitivity underscores the importance of balancing monomer reactivity and mechanical performance to ensure structural stability during and after printing. Previous studies have reported varying formulations across this compositional spectrum. For instance, ref. [13] achieved successful printing with a VP: PEGDA ratio of 70:30 (*w*/*w*) using Phenylbis (2,4,6-trimethylbenzoyl)phosphine oxide (BAPO) as a photoinitiator, while [18] employed an aqueous formulation containing 5 wt% PEGDA, 10 wt% VP, and 1 wt% LAP. Furthermore, ref. [12] demonstrated a workable LAP concentration range between 0.23 and 0.49% (*w*/*v*) for comparable PEG-based systems, highlighting the interplay between photoinitiator loading and polymer conversion efficiency. LAP exposures up to 0.5 wt% without light have been reported as non-cytotoxic [24].

Building on these findings, a PEGDA/VP ratio of 40:60 (%*w*/*w*) was identified as optimal in this work, yielding consistent vat recoating behaviour and high-fidelity layer formation within the DLP vat photopolymerisation process. At this composition, the resin maintained a manageable viscosity, ensuring uniform layer deposition and preventing film defects during printing. Photoinitiator concentration screening within the range of 0.1–0.5 wt% LAP revealed that 0.4 wt% LAP achieved the best balance between curing speed and structural precision. At this loading, the resin exhibited rapid polymerisation with complete crosslinking in the expected exposure time and light intensity, minimising cure-through while ensuring adequate interlayer adhesion. Overall, the optimised PEGDA/VP (40:60) resin with 0.4 wt% LAP demonstrated excellent processability, reproducibility, and structural integrity, establishing a reliable photopolymer matrix for DLP-based fabrication of DMNs.

### 3.3. Rheological Characterisation of the Ink

The printability of photopolymerizable resin was largely influenced by its viscosity. It was reported that for resin-based 3D printers (SLA and DLP), the ideal viscosity of resin should be up to 10^3^ mPa.s with a shear rate of 80–100 s^−1^ [19,25]. Resin with high viscosity presented challenges during printing, while the formation of layer-by-layer procedure, specifically in adhering to the build platform for the 3D printed objects. Consequently, resin with relatively low viscosity facilitated the efficient and precise manufacturing of object formulation. In DLP printing, the resin liquid sample is typically subjected to a shear rate ranging between 80 and 100 s^−1^. This applied shear rate played an essential role because it influenced the flow and levelling of resin, ensuring smooth layer formation and precise printing.

**Figure 3:**
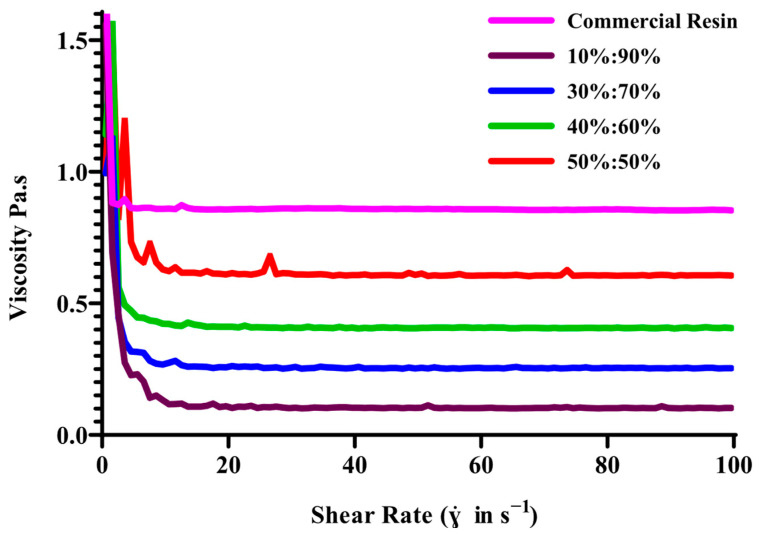
Viscosity profiles of PEGDA/VP resin formulations with varying polymer ratios under continuous shear-rate sweep (0–100 s^−1^, *n* = 3).

The study investigated and confirmed that the resin formulations exhibit stable and consistent viscosity values, making them suitable for DLP 3DP. This stability was crucial for the precise and reliable production of MN patches, ensuring predictable behaviour throughout the printing process. The combination of PEGDA and VP in a 40:60% *w*/*w* ratio displayed ideal properties for the MNs printing process, achieving the precise balance needed for effective layer-by-layer formation via DLP printing. The study revealed that the viscosity of the resin mixture was influenced by the properties of PEGDA used in conjunction with VP, with increasing PEGDA concentration resulting in higher viscosity. This combination played a significant role in maintaining vital viscosity values throughout the process. Resins exhibited shear-thinning behaviour and acted as Newtonian liquids, maintaining a plateau phase concerning increased shear rates shown in Figure 3. Although the custom 40:60 PEGDA/VP ink exhibited exceptionally lower viscosity than commercial PlasCLEAR^®^ resin, it nonetheless delivered excellent printability and shape fidelity. Notably, resin formulations with PEGDA/VP ratios of 10:90 and 30:70 exhibited comparable rheological characteristics. In case of 50:50 formulation viscosity values comparable to the commercial reference resin; its higher PEGDA content led to incomplete recoating and reduced curing uniformity during DLP printing. However, a comprehensive evaluation of print fidelity and monomer conversion demonstrated that a 40:60 PEGDA/VP mixture achieved the most favourable balance between viscosity and curing efficiency. This optimised ratio enabled superior layer recoating and near-complete monomer conversion, as evidenced in the proton nuclear magnetic resonance detection of residual monomers. Consequently, the 40:60 PEGDA/VP formulation was selected for subsequent DMN fabrication owing to its enhanced printability and mechanical robustness.

### 3.4. Fabrication of MN Arrays via DLP 3D Printing

**Figure 4:**
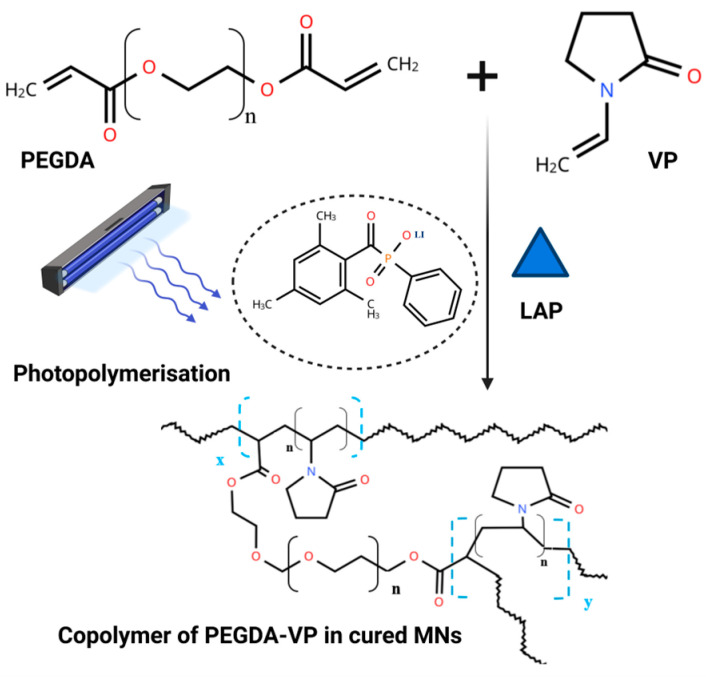
Schematic representation of the photopolymerisation process during DLP-based MN fabrication.

The DLP 3D printing process was optimised to fabricate MN arrays with high dimensional accuracy and mechanical integrity. Printing parameters were carefully tuned to establish a reliable formulation-process window for the photopolymerisable resin system composed of PEGDA, VP, and LAP (Figure 4). An irradiance of 26.61 mW cm^−2^ with a layer exposure time of 3 s consistently produced MN arrays with reproducible geometries, sharp tips, and uniform base formation. These parameters provided sufficient energy for complete layer crosslinking while avoiding over-curing or interlayer fusion artefacts.

The resin composition strongly influenced print fidelity and mechanical strength. PEGDA acts as a crosslinking monomer, imparting elasticity and dimensional stability, whereas VP contributes to rapid photopolymerisation and rigidity upon curing. PEGDA-rich blends yielded softer structures prone to deformation, while VP-rich systems were brittle and susceptible to cracking, findings consistent with earlier reports highlighting the need for balanced reactivity between hydrophilic monomers [13,18]. This brittleness arises because VP polymerises to form PVP segments with high glass-transition temperatures; the resulting networks have high crosslink density and limited chain mobility, which increases stiffness but reduces toughness. In contrast, PEGDA introduces flexible poly (ethylene glycol) linkages that lower the network modulus and allow stress dissipation. Therefore, excessive VP content produces rigid, brittle resins susceptible to cracking, whereas higher PEGDA content yields softer but more resilient structures [13]. An optimal PEGDA-to-VP ratio is thus crucial to ensure sufficient rigidity for insertion while avoiding brittle fracture. The optimised composition, PEGDA/VP at 40:60 *w*/*w* with 0.4 wt% LAP, achieved efficient photopolymerisation within 3 s per layer, producing defect-free arrays with stable interlayer bonding. This concentration of LAP provided a rapid cure rate without inducing cure-through or feature broadening, which can otherwise arise from excessive photoinitiator loading [13]. The selected formulation exhibited a viscosity plateau near 0.1 Pa·s at 25 °C, facilitating uniform resin recoating and smooth layer transitions during printing. Arrays fabricated under these conditions displayed consistent MN height and base dimensions, closely matching their CAD models, as evidenced in the morphological and physical characterisation of MN arrays. No significant morphological defects such as tip rounding, warping, or resin pooling were observed, confirming that the chosen irradiance and exposure duration effectively confined polymerisation to intended voxel boundaries. The optimised parameters yielded reproducible MN arrays with the precision and structural resilience necessary for downstream testing in insertion, dissolution, and swelling studies.

### 3.5. Fourier-Transform Infrared Spectroscopic Analysis

FTIR spectroscopy was used to investigate the chemical structure of the photopolymerised MN arrays and to confirm the conversion of monomers to a crosslinked polymer network. The analysis aimed to (i) compare the spectra of the cured MNs with those of the neat resin components (PEGDA, VP, and LAP) and (ii) elucidate potential intermolecular interactions within the final polymeric matrix. The FTIR spectrum of PEGDA exhibited characteristic absorption bands at 2889 cm^−1^ (asymmetric CH_2_ stretching), 1721 cm^−1^ (acrylate C=O stretching), and 1623 cm^−1^ (aliphatic C=C stretching), along with peaks at 1110, 960, and 843 cm^−1^ assigned to C–O stretching and CH_2_=CH out-of-plane vibrations [26]. The spectrum of VP showed a prominent lactam carbonyl absorption near 1750 cm^−1^, C–H stretching vibrations at 2981 and 2889 cm^−1^, an aliphatic C=C band around 1634 cm^−1^, methylene bending at 1422 and 1382 cm^−1^, and a C–N stretching band near 1264 cm^−1^. An N–H stretch was observed at 3474 cm^−1^, and a distinct band at approximately 845 cm^−1^ corresponded to =C–H modes [27]. Following photopolymerisation, the FTIR spectrum of the printed MNs revealed marked spectral transformations relative to the monomeric components. The vinylic C=C absorption at ~1640 cm^−1^ was significantly reduced or absent, indicating extensive double-bond consumption and successful polymer network formation. The carbonyl stretching region broadened between 1660 and 1740 cm^−1^, reflecting the superimposed contributions of the PEGDA ester and VP lactam carbonyl groups, as well as possible hydrogen-bonding interactions between them. A strong absorption band in the 1000–1040 cm^−1^ range, attributed to C–O–C stretching, further confirmed the formation of the PEGDA-based ether linkages within the crosslinked backbone [28]. No discernible peaks corresponding to the photoinitiator (LAP) were detected, which is consistent with its low concentration (0.4 wt%) relative to the polymer matrix and with the initiator residues remaining spectroscopically silent at such loadings. The spectral convergence of the PEGDA and VP bands in the polymerised sample indicates successful integration of both monomers into a homogeneous copolymeric network.

**Figure 5:**
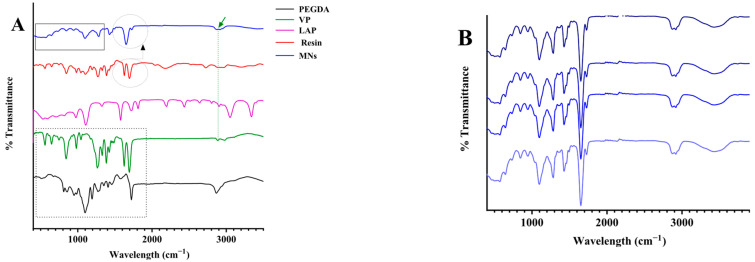
ATR-FTIR spectral analysis confirming polymerisation and chemical uniformity of MN arrays. (**A**) Spectra of PEGDA, VP, LAP, the uncured resin, and the photopolymerised MNs (the dotted rectangle, dashed circle, and vertical line highlight characteristic functional groups). (**B**) FTIR spectra collected from different regions of the MNs matrix.

Collectively, these findings confirm efficient photopolymerisation under the optimised DLP printing conditions and substantiate the establishment of a stable PEGDA/VP interpenetrating network. The disappearance of the C=C band, along with the broadening of carbonyl and ether absorptions, provides direct spectroscopic evidence for extensive monomer conversion and complete network formation. These spectral transformations validate the chemical integrity of the printed MNs and confirm that the polymerisation proceeded uniformly throughout the structures. The key peak assignments supporting this interpretation are summarised in Table 2, while representative spectra of the resin components and printed MNs are presented in Figure 5.

### 3.6. Proton Nuclear Magnetic Resonance Detection of Residual Monomers

**Figure 6:**
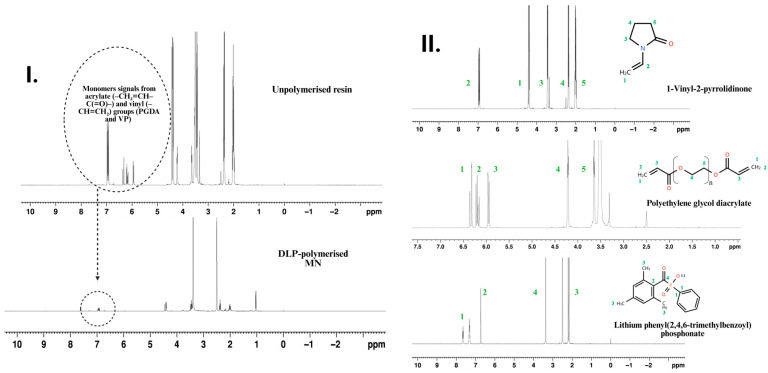
^1^H NMR spectroscopic confirmation of monomer conversion and structural assignment in the PEGDA/VP photo-resin and printed MN arrays. (**I**) Comparative spectra of the uncured PEGDA/VP resin and the DLP-polymerised MNs. (**II**) ^1^H NMR spectra of individual resin components, VP, PEGDA, and LAP. Coloured numbering on the spectra denotes the proton assignments as indicated on the corresponding chemical structures.

Quantitative ^1^H Nuclear Magnetic Resonance (NMR) spectroscopy was employed to evaluate monomer conversion and the presence of residual extractables within the PEGDA/VP photo-resins and their corresponding MN arrays (Figure 6). The analysis provided a molecular-level confirmation of polymerisation efficiency achieved under the optimised DLP printing parameters. Distinct vinyl proton signals from PEGDA (δ 5.9–6.4 ppm) and from vinylpyrrolidone (δ ≈ 6.9 ppm) were used as diagnostic markers of unreacted monomers [13]. In the uncured PEGDA/VP (70:30 *w*/*w*) formulation, these resonances were clearly visible alongside the characteristic aliphatic backbone peaks, confirming the presence of polymerisable C=C functionalities. In contrast, the spectra obtained from the printed MN arrays exhibited complete disappearance of all vinylic signals, which fell below the baseline noise level, indicating nearly quantitative consumption of double bonds throughout the three-dimensional structure. Integration of residual signals relative to the polymer backbone protons allowed estimation of unreacted monomer content below 0.5%, a value well beneath the screening threshold generally cited for polymeric medical devices. Such low extractable levels are consistent with previously reported PEG-based photopolymer networks demonstrating excellent cytocompatibility and negligible leachability under physiological conditions [13].

The disappearance of vinylic resonances corroborates the FTIR evidence of C=C bond depletion and supports the high crosslinking efficiency inferred from thermal and mechanical data. Collectively, these findings confirm that the selected formulation (PEGDA/VP 40:60 *w*/*w*; 0.4 wt% LAP) and exposure regime (26.61 mW cm^−2^ × 3 s per layer) produced a fully polymerised, chemically stable matrix essentially free of unreacted acrylate or vinyl species. This high degree of cure ensures structural integrity and minimises the risk of cytotoxicity.

### 3.7. Thermal Analysis

#### 3.7.1. Thermogravimetric Analysis

TGA was performed on the individual polymers (PEGDA, VP, and LAP), the uncured PEGDA/VP resin, and the DLP-printed MN arrays (*n* = 3 per sample) to evaluate their thermal behaviour and to verify polymer network formation. Although fabrication was conducted at ambient temperature, well below the degradation thresholds of all components, TGA served as an indirect probe of polymerisation by monitoring changes in the degradation profile from feed materials to the final printed constructs. The uncured PEGDA/VP resin exhibited a characteristic two-step mass-loss pattern upon heating. The first weight-loss event, initiating near 100 °C, corresponded to volatilisation or thermal decomposition of the VP component, while the second, more pronounced event at approximately 400 °C was associated with the decomposition of PEGDA. This multi-stage degradation reflects the heterogeneous composition of the uncured resin and the limited molecular interaction between its components prior to photopolymerisation. In contrast, the printed MNs demonstrated a markedly improved thermal stability, displaying a single major degradation step beginning beyond 420 °C. The suppression of the early mass-loss event and the upward shift in the principal degradation temperature relative to the uncured resin indicate successful crosslinking of VP and PEGDA into a stable polymeric network. This enhancement in thermal resistance can be attributed to the immobilisation of VP chains within the crosslinked PEGDA matrix, which restricts volatilisation and raises the energy barrier for decomposition.

**Figure 7:**
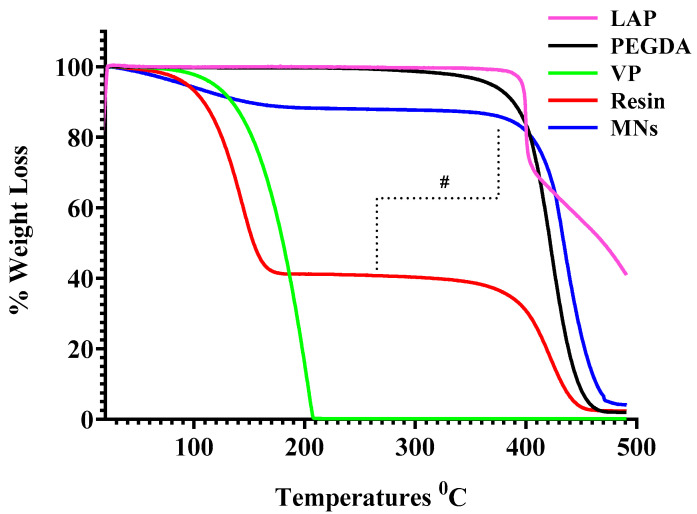
TGA of the individual polymers (PEGDA, VP, and LAP), the uncured PEGDA/VP resin, and the DLP-printed MN arrays. “#” indicates the two-step thermal degradation observed in the uncured resin, in contrast to the single, sharp thermal degradation event exhibited by the crosslinked MN, reflecting network stabilisation after photopolymerisation.

These findings align with the expected behaviour of photo-crosslinked PEGDA-based systems, where network formation increases molecular weight, reduces free-monomer mobility, and improves thermal endurance. A minor mass loss (~10%) observed up to ~150 °C for the printed arrays () is attributed to the release of absorbed moisture and residual low-molecular-weight species entrapped within the hydrophilic PEGDA/VP network; such dehydration is typical for crosslinked hydrogel matrices and highly hydrophilic polymers and does not imply the presence of unreacted VP. Similar TGA studies report that hydrophilic polymers lose mass at 50–160 °C due to the removal of free and capillary moisture [29], and that PVP samples show ~9% weight loss below 250 °C solely from moisture desorption [30]. This early weight loss is therefore consistent with the nearly quantitative consumption of C=C bonds evidenced by FTIR and DSC analyses. The observed thermal profile corroborates the FTIR and NMR data, collectively confirming complete polymerisation and the formation of a chemically stable matrix suitable for downstream processing and application. Representative thermograms of all materials are presented in Figure 7, highlighting the comparative improvement in thermal stability achieved upon DLP-induced crosslinking.

#### 3.7.2. Differential Scanning Calorimetry

**Figure 8:**
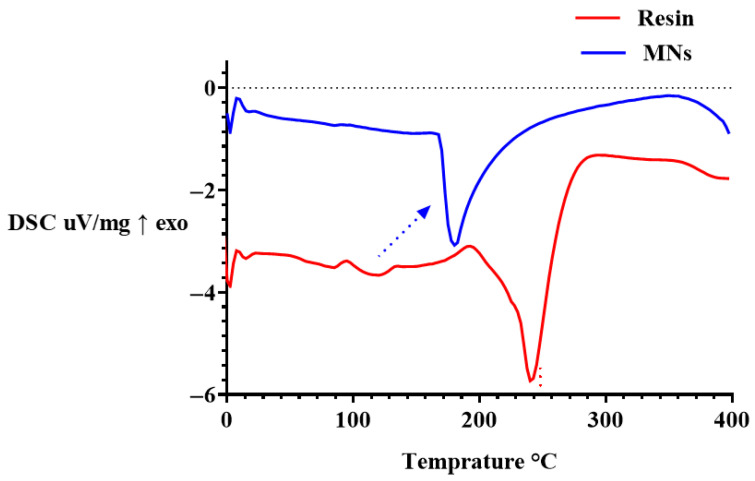
DSC thermograms of the uncured PEGDA/VP resin and DLP-printed MN arrays (exo ↑). The red dotted line indicates the principal degradation transition of the uncured resin, while the blue arrow highlights a minor relaxation event (~180 °C) in the crosslinked MNs. The upward shift in the main endothermic transition in the MNs demonstrates enhanced thermal stability following photopolymerisation.

DSC was performed to evaluate the thermal transitions and phase behaviour of the uncured PEGDA/VP resin and the corresponding DLP-printed MN arrays (Figure 8). The thermograms provide insight into how photopolymerisation alters polymer chain dynamics and network organisation within the final constructs. The uncured resin exhibited a prominent endothermic transition between approximately 200 °C and 300 °C, which can be attributed to the onset of thermal degradation of the PEGDA/VP mixture rather than polymer softening, as the precursor contains oligomeric PEGDA and monomeric VP. This event aligns with the main mass-loss step seen in TGA, which appears at slightly higher temperature due to the faster DSC heating rate. In contrast, the printed MNs displayed a single, attenuated endothermic event centred around ~180 °C with markedly reduced enthalpy, followed by a major transition shifted towards higher temperature, confirming greater thermal stability of the crosslinked network. The small endothermic feature at ~180 °C in the printed MNs may be associated with structural relaxation or rearrangement within the crosslinked PEGDA/VP network before thermal degradation, consistent with the absence of melting peaks of unreacted species in FTIR and TGA results. The upward shift and broadening of this transition indicate restricted chain mobility and increased structural rigidity, consistent with the formation of a covalently crosslinked PEGDA-VP network during DLP printing. The reduction in transition intensity and absence of additional endothermic peaks below 200 °C confirm the absence of residual unreacted monomers or phase-separated domains within the cured MNs. These findings agreed with previous reports describing similar thermal signatures for crosslinked PEGDA-based MNs [18]. The absence of new thermal events below 200 °C also implies that the printed arrays remain stable under ambient and physiological temperature ranges, ensuring material robustness during handling and application.

Overall, the DSC results demonstrate that photopolymerisation significantly modifies the thermal response of the resin by forming a rigid, crosslinked polymer network while preserving overall thermal stability. The observed transition behaviour supports the conclusions drawn from FTIR, NMR, and TGA analyses, collectively confirming complete polymerisation and enhanced structural integrity of the fabricated MNs.

### 3.8. Morphological and Physical Characterisation of MN Arrays

**Figure 9:**
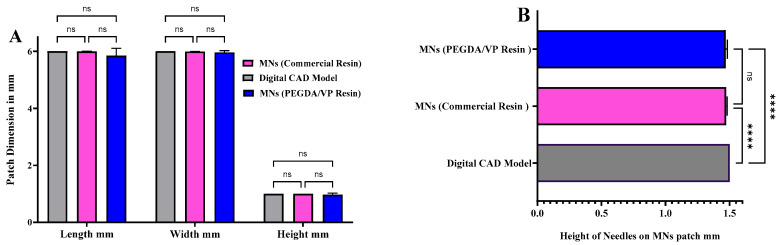
(**A**) Comparison of patch dimensions (length, width, and height) among the digital CAD model, commercial-resin microneedles, and PEGDA/VP-based microneedles showing no significant difference (ns, *p* > 0.05). (**B**) Comparison of individual microneedle heights from CAD model, commercial resin, and PEGDA/VP MNs. A slight but statistically significant difference (****, *p* < 0.0001) was observed between the printed arrays and the CAD reference, while no difference (ns) was noted between the two printed groups. Data are expressed as mean ± SD, *n* = 3.

To ensure geometrical accuracy of the printed arrays, optical microscopy was employed to assess dimensional fidelity relative to the original CAD models. Digital light microscopic images of all seven designs are illustrated in Figure S1 (Supplementary Data). Quantitative analysis was conducted on MNs fabricated from the optimised PEGDA/VP formulation (40:60 *w*/*w* with 0.4 wt% LAP) and compared with arrays printed using a commercial reference resin. As shown in Figure 9A, the measured patch dimensions (length, width, and overall thickness) for both the PEGDA/VP-based and commercial-resin MNs closely matched the corresponding CAD model, with no statistically significant differences (*p* > 0.05, ns). The high degree of dimensional agreement demonstrates precise layer stacking and controlled polymerisation throughout the DLP printing process. Similarly, the individual needle heights presented in Figure 9B remained consistent between the two resin systems, exhibiting no measurable deviation from the intended 1.5 mm CAD specification (ns).

However, when compared directly to the digital CAD reference, both printed MN sets showed a minor but statistically significant reduction in average height (**** = *p* < 0.0001), attributed to controlled optical penetration limits and layer-by-layer shrinkage during photopolymerisation. Despite this slight contraction, the overall morphological accuracy reached 98.6 ± 0.5%, confirming excellent geometric fidelity across all builds. The optical micrographs confirmed sharp tip formation, uniform base profiles, and absence of voids or delamination, supporting consistent print reproducibility. Accurate replication of CAD geometry is essential to ensure predictable insertion behaviour and reproducible skin engagement.

**Figure 10:**
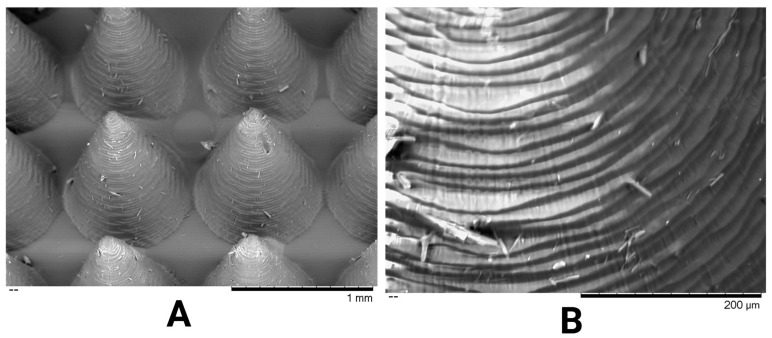
SEM micrographs of DLP-printed PEGDA/VP MN arrays. (**A**) Low-magnification image showing uniform conical needles with smooth alignment and consistent spacing across the patch (scale bar = 1 mm). (**B**) High-magnification image highlighting the layer-by-layer surface texture characteristic of the DLP process and sharply defined needle tips (scale bar = 200 µm).

SEM was employed to evaluate the surface morphology, tip geometry, and microstructural integrity of the MN arrays. Representative SEM micrographs are shown in Figure 10, providing both low- and high-magnification perspectives of the printed needles. At low magnification (Figure 10A), the MN array exhibited uniform, axially symmetric conical structures with sharply defined apices and consistent inter-needle spacing across the patch surface, indicating excellent printing fidelity and reproducibility. The uniformity of the array confirms accurate photopolymerisation throughout the DLP process, with no evidence of tip deformation, void formation, or base delamination. Higher-magnification imaging (Figure 10B) revealed the presence of fine concentric striations along the needle shafts, characteristic of the layer-by-layer curing inherent to the DLP technique. These microscale surface undulations are not indicative of print defects but reflect the photopolymerisation steps that define voxel-layer stacking. Such subtle surface roughness may enhance mechanical interlocking with the stratum corneum during insertion and potentially improve wetting and dissolution dynamics once in contact with interstitial fluid. Tip sharpness was consistently achieved across the examined arrays, with an average tip diameter of 86.35 ± 3.01 µm. Overall, SEM analysis demonstrated high geometric precision and superior surface quality of the printed MNs. Also, to verify batch uniformity as physical properties, MN arrays were individually weighed (*n* = 3), showing minimal mass variation (SD = 0.52 mg). This high manufacturing consistency further confirms the reproducibility of the DLP-based fabrication process.

### 3.9. Mechanical and Insertion Performance of MNs

#### 3.9.1. Parafilm^®^-Based Insertion Analysis

**Figure 11:**
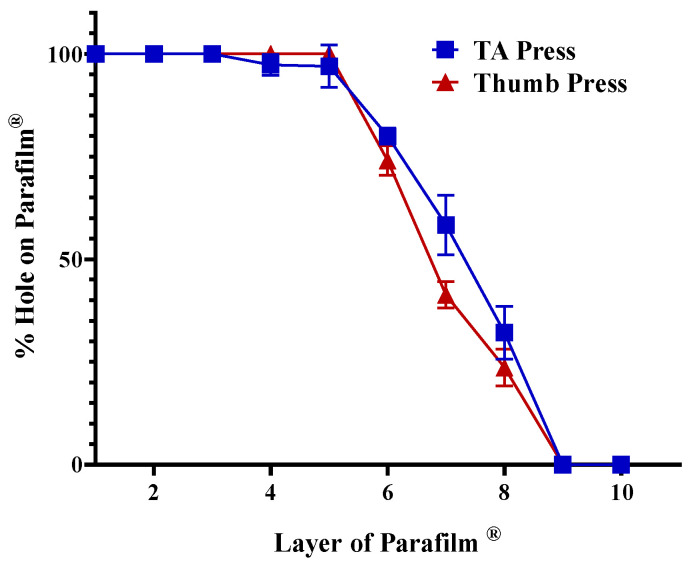
Insertion efficiency of MN arrays evaluated using Parafilm^®^ M multilayer model under TA and thumb-press conditions. Data represent SD (*n* = 3).

The insertion capability of the printed MN arrays was initially evaluated using the multilayer Parafilm^®^ M model to simulate skin penetration mechanics. Instrumented testing was performed with a texture analyser at a compression speed. The percentage of holes formed in each Parafilm^®^ layer was used to quantify insertion depth. As shown in Figure 11, the MN arrays achieved 100% perforation through the upper five layers, equivalent to a penetration depth of approximately 0.6 mm, comparable to full stratum corneum and viable epidermis thickness. A parallel thumb-press test yielded similar results, demonstrating nearly complete perforation across four Parafilm^®^ layers with ≤0.5% reduction in needle height, indicating minimal plastic deformation. These outcomes confirm that the printed MNs possess sufficient mechanical strength to breach surrogate skin barriers under both instrumented and manual loading conditions. The near-identical trends between the texture analyser and thumb-press curves further validate their practical robustness for user-applied administration.

#### 3.9.2. Ex Vivo Insertion on Porcine Skin and Chicken Breast Models

**Figure 12:**
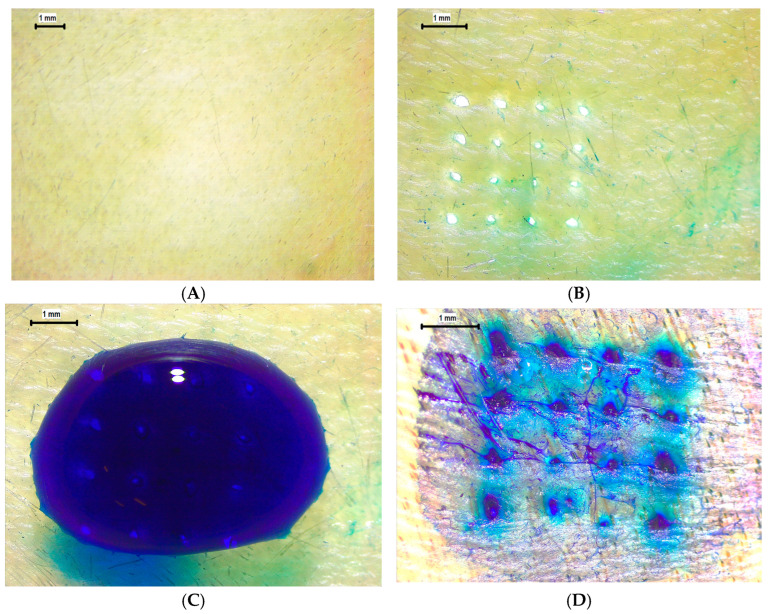

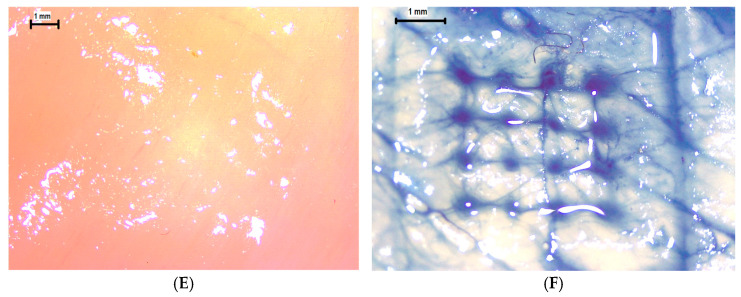
Insertion and ex vivo penetration evaluation of MNs. (**A**) Porcine skin before insertion (10×). (**B**) Porcine skin after insertion (16×). (**C**) Application of methylene blue (16×). (**D**) Formation of dye-filled insertion holes (25×). (**E**) Chicken breast before insertion (10×). (**F**) Chicken breast after insertion showing distinct punctures (16×).

To further assess insertion efficacy under more physiologically relevant conditions, ex vivo evaluations were performed using neonatal porcine skin and chicken breast (pectoralis major) tissues. On porcine skin, the pre-application surface (Figure 12A) appeared intact and unbroken, whereas post-insertion imaging (Figure 12B) revealed clean, discrete punctures corresponding precisely to the 4 × 4 MN array pattern. Following methylene blue application (Figure 12C), distinct dye-filled microchannels confirmed successful skin penetration and channel patency. The dye diffusion around each insertion site (Figure 12D) indicates partial lateral permeation, consistent with microchannel interconnection within the dermal layer. For the chicken breast model, similar patterns were observed: the surface before application (Figure 12E) was smooth and unaltered, while post-application images (Figure 12F) demonstrated uniform puncture formation with no evidence of tip bending, shank buckling, or material fracture. These findings confirm that the printed MNs maintain mechanical integrity and sharpness during insertion into both compliant (chicken and porcine) tissues.

Collectively, the Parafilm^®^ data and ex vivo observations confirm that the optimised PEGDA/VP microneedles achieve high insertion efficiency with minimal deformation, ensuring reliable skin engagement.

### 3.10. Dissolution Behaviour, Swelling Dynamics, and Degradation Analysis of MNs

#### 3.10.1. Dissolution Behaviour

DMN formulations rely on rapid hydration and dissolution of the polymer matrix to release their payload into the skin. In this study, dissolution was evaluated in both de-ionised water and phosphate-buffered saline (PBS, pH 7.4) using whole MN patches and individual needles excised from the patch. Both media supported dissolution of the PEGDA/VP arrays, but the kinetics differed. When intact patches were immersed in water, the hydrophilic vinyl-pyrrolidone component readily absorbed fluid, leading to disintegration of the matrix within a few hours. In PBS, whole patches initially swelled and began to lose mass; they disintegrated over ~5 h and completely dissolved overnight with minimal residue. In contrast, individual needles dissolved much more rapidly in PBS, completely disappearing after ~5 h. PVP is known to be water-soluble and biocompatible; when incorporated into microneedles, it absorbs interstitial fluid and promotes polymer dissolution [31]. The rapid disappearance of the isolated needles supports efficient dissolution of the VP-rich network and indicates that the bulk patch thickness governs the overall dissolution time. These results confirm that the PEGDA/VP DMN system possesses adequate solubilisation capacity for transdermal drug delivery.

#### 3.10.2. Swelling Study

**Figure 13:**
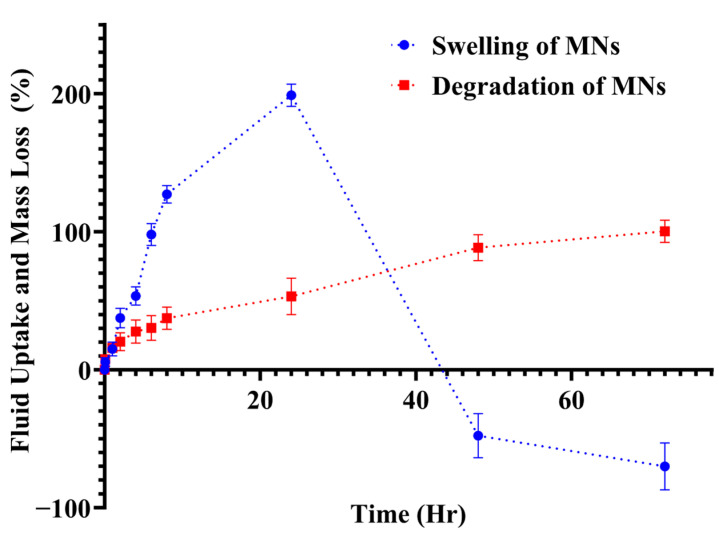
Fluid uptake (swelling) and mass loss (degradation) of PEGDA/VP microneedle patches in PBS (pH 7.4). Swelling (blue circles) shows rapid hydration during the first hour followed by continued uptake over ~5 h, while degradation (red squares) indicates gradual mass loss progressing to ~98% by 72 h. Values represent mean ± SD (*n* = 3).

Swelling behaviour was characterised by immersing MN patches in PBS (pH 7.4) at 32 ± 0.5 °C and measuring mass gain at predefined intervals. The optimised PEGDA/VP composition showed pronounced swelling during the first hour, followed by continued uptake over the next ~5 h (Figure 13). This rapid initial hydration can be attributed to the hydrophilic nature of VP, which readily absorbs aqueous media and promotes water ingress into the polymer network. The time-dependent swelling profile suggests two distinct phases: an initial “priming” phase in which water rapidly infiltrates the MNs shafts and patch matrix, and a slower phase where continued hydration leads to moderate additional mass gain. After ~24 h, the percentage swelling decreased slightly as erosion of the cross-linked matrix commenced. Comparable PEGDA/VP systems have shown that the swelling ratio and mesh size depend on crosslink density and UV exposure time; higher crosslink density reduces swelling but increases network stability [32]. The present formulation displayed a more pronounced early swelling than previously reported PEGDA/VP compositions [18], likely due to the higher VP fraction and lower crosslink density. This swelling behaviour may create a window for active molecule release: a rapid hydration phase that can facilitate immediate exposure of the loaded actives at the skin interface, followed by a slower erosion phase that sustains release over several hours.

#### 3.10.3. Degradation Analysis

Gravimetric degradation studies were conducted concurrently with the swelling experiments to quantify mass loss of MN patches over 72 h. The PEGDA/VP networks showed a progressive increase in mass loss, reaching approximately 98 ± 1.05% by 72 h (Figure 13). Visual inspection revealed gradual rounding of patch edges, reduction in surface relief and eventual disappearance of MN features, indicating erosion of the polymer matrix. The degradation mechanism is consistent with hydrolytic cleavage of the acrylate ester end groups and gradual dissolution of the crosslinked PEGDA/VP network. PEGDA hydrogels degrade predominantly via hydrolysis of end-group esters and, depending on macro molecular weight and crosslink density, can take months to years in vivo [32]. The faster degradation observed in this study reflects the relatively low crosslink density and the presence of hydrophilic VP, which accelerates water uptake and polymer dissolution. By adjusting the PEGDA/VP ratio and the photoinitiator concentration, it should be possible to tailor the degradation rate and thus control the duration of release. The combination of swelling data and mass-loss profiles, therefore, may provide a rational basis for engineering DMN formulations that could deliver an initial burst of therapeutic agent followed by sustained release as the matrix erodes.

### 3.11. Surface Wettability Assessment by Contact Angle Goniometry

The surface wettability of the MN patches was quantified via static contact-angle measurements using the sessile-drop method. A 5 µL deionised water droplet was deposited onto the flat surface of the PEGDA/VP patch (excluding the needles), and the droplet profile was recorded at ambient conditions. The static contact angle was measured over 60 s and averaged, yielding a value of 35.19 ± 7.08° (*n* = 3) in Figure S2. According to established definitions of wettability, water contact angles below 90° denote hydrophilic surfaces, whereas angles above 90° indicate hydrophobicity; thus, the printed patches are clearly hydrophilic.

This hydrophilic behaviour can be attributed to the high VP content of the PEGDA/VP formulation. A moderate contact angle (≈35°) represents a favourable balance: it is low enough to ensure rapid spreading of interstitial fluid across the patch surface during application, facilitating swift hydration and swelling of the microneedle matrix, yet not so low as to cause premature dissolution during handling. In the context of dissolving microneedles, such wettability is beneficial because it supports initial fluid uptake while maintaining structural integrity until insertion. The measured value therefore confirms that the optimised PEGDA/VP composition provides a surface chemistry conducive to efficient swelling and dissolution without compromising manufacturability.

## 4. Conclusions

This work demonstrates that a PEGDA/VP photo-resin, processed via pixel-aware DLP, can produce DMN arrays with high geometric fidelity and reproducible mechanics. The optimised formulation (40 wt% PEGDA, 60 wt% VP, 0.4 wt% LAP) cured rapidly and yielded arrays whose dimensions matched their CAD models. CAG revealed a hydrophilic surface (~35°), which is conducive to rapid wetting. Mechanical insertion tests showed reliable penetration: Parafilm^®^ stacks exhibited 100% perforation through five layers, and thumb-press tests achieved complete insertion through four layers; ex vivo models showed clean punctures without fracture. Dissolution and swelling studies highlight a two-phase release profile: individual needles dissolved fully within ~5 h in pH 7.4 buffer, while whole patches disintegrated by ~4 h and dissolved overnight; swelling kinetics displayed rapid fluid uptake in the first hour followed by gradual erosion, and gravimetric analysis indicated ~85% mass loss after 72 h. Thermal and spectroscopic analyses confirmed extensive polymerisation and structural stability, and post-insertion height change remained ≤0.5%. In summary, the results demonstrate that pixel-aware DLP processing of PEGDA/VP resin yields DMN arrays with high geometric fidelity, robust mechanical performance and controlled dissolution behaviour, which may have promising attributes for transdermal drug delivery. Additionally, the formulation may serve as a promising carrier for future biologics and drug-loaded MN systems aimed at effective and patient-friendly transdermal delivery.

## Figures and Tables

**Table 1:** Structural specifications of the MN array designs modelled using Tinkercad.

S.N.	Dimensions (mm)			Number of Needles	Geometry Type Patch
	Patch (LWH)	Needle Height	Needle Base Radius		
1	14 × 14 × 1	1.3	1.0	16	Pyramidal (Square)
2	12 × 12 × 1.5	1.3	0.9	20	Conical (Square)
3	12 × 12 × 0.5	1.0	0.5	50	Conical (Circle)
4	10 × 10 × 1	1.5	0.3	16	Conical (Square)
5	10 × 10 × 1	1.5	0.3	36	Conical (Square)
6	8 × 8 × 2	1.0	0.4	21	Conical (Circle)
7	6 × 6 × 1	1.5	0.6	16	Conical (Square)

**Table 2:** Characteristic FTIR absorption bands and corresponding functional-group assignments for PEGDA, VP, LAP, and the printed MNs.

Materials	Functional Group	Wavelength cm^−1^	Reference
VP	−C stretching of the five-membered cyclic lactam structure	1750	-
	C=C stretching of unsaturated groups (alkenes)	1634	
	C–H bending vibrations from CH_3_ groups	1422–1382	[27]
	=C−H stretching and bending vibrations	845	
PEGDA	CH_2_ asymmetric stretch vibration	2889	[26]
	C=O vibrations in acrylates	1721	
	C=C vibration of the aliphatic double bond	1623	
	C–O stretch vibration	1110	-
	CH_2_=CH symmetrical stretching and vibration	960, 843	
	C=N and C=C stretching vibration aromatic ring	1429–1660	
	vibration of the pyrimidine ring	1348	
	C−O and C−N stretching vibrations	1180	
	C−N stretching vibrations	1246	
MNs	Overlapping ester carbonyl and C−N stretching vibrations	1660 to 1740	[18,28]
	C−O−C linkage	1000–1040	-
	C=C aromatic stretching	1454	-

## Data Availability

The raw data supporting the conclusions of this article will be made available by the authors on request.

## Supplementary Materials

The following supporting information can be downloaded at: https://www.mdpi.com/article/10.3390/pharmaceutics17111498/s1, Figure S1: Images of DLP-MN arrays fabricated using the optimised PEGDA/VP (40:60 *w*/*w*) resin formulation.; Figure S2: Static contact angle measurement of the PEGDA/VP microneedle patch using the sessile-drop method.

## Abbreviations

^1^H NMR	Proton Nuclear Magnetic Resonance
ANOVA	Analysis of Variance
ATR-FTIR	Attenuated Total Reflectance Fourier-Transform Infrared
CAD	Computer-Aided Design
CAG	Contact Angle Goniometry
DMNs	Dissolving Microneedles
DLP	Digital Light Processing
DMSO-d_6_	Deuterated Dimethyl Sulfoxide
DSC	Differential Scanning Calorimetry
FTIR	Fourier-Transform Infrared Spectroscopy
FIB-SEM	Focused Ion-Beam Scanning Electron Microscopy
LAP	Lithium Phenyl(2,4,6-Trimethylbenzoyl) phosphinate
MN	Microneedle
MNs	Microneedles
NA	Not Applicable
PBS	Phosphate-Buffered Saline
PEGDA	Polyethylene Glycol Diacrylate
PVP	Polyvinylpyrrolidone
SEM	Scanning Electron Microscopy
TA	Texture Analyser
TGA	Thermogravimetric Analysis
TMS	Tetramethylsilane
UV	Ultraviolet
VP	Vinylpyrrolidone
3DP	Three-Dimensional Printing
