# Supplementary material for: Mould-Free Microneedles in a Single Step: 3D Printing with Photopolymer Resins for Transdermal Delivery
Source: Pharmaceutics. 2025 Nov 19;17(11):1498. doi: 10.3390/pharmaceutics17111498 (PMC12655701; doi:10.3390/pharmaceutics17111498)
Supplement: Supplementary file 1 [file pharmaceutics-17-01498-s001.zip › pharmaceutics-3981538-supplementary.pdf]

pharmaceutics

MDPI

Supplementary Material

Mould-Free Microneedles in a Single Step: 3D Printing with Photopolymer Resins for Transdermal Delivery

Rutuja N. Meshram and Dimitrios A. Lamprou *

School of Pharmacy, Queens University Belfast, 97 Lisburn Road, Belfast BT9 7BL, UK; rmeshram01@qub.ac.uk

* Correspondence: d.lamprou@qub.ac.uk; Tel.: +44-(0)28-9097-2617

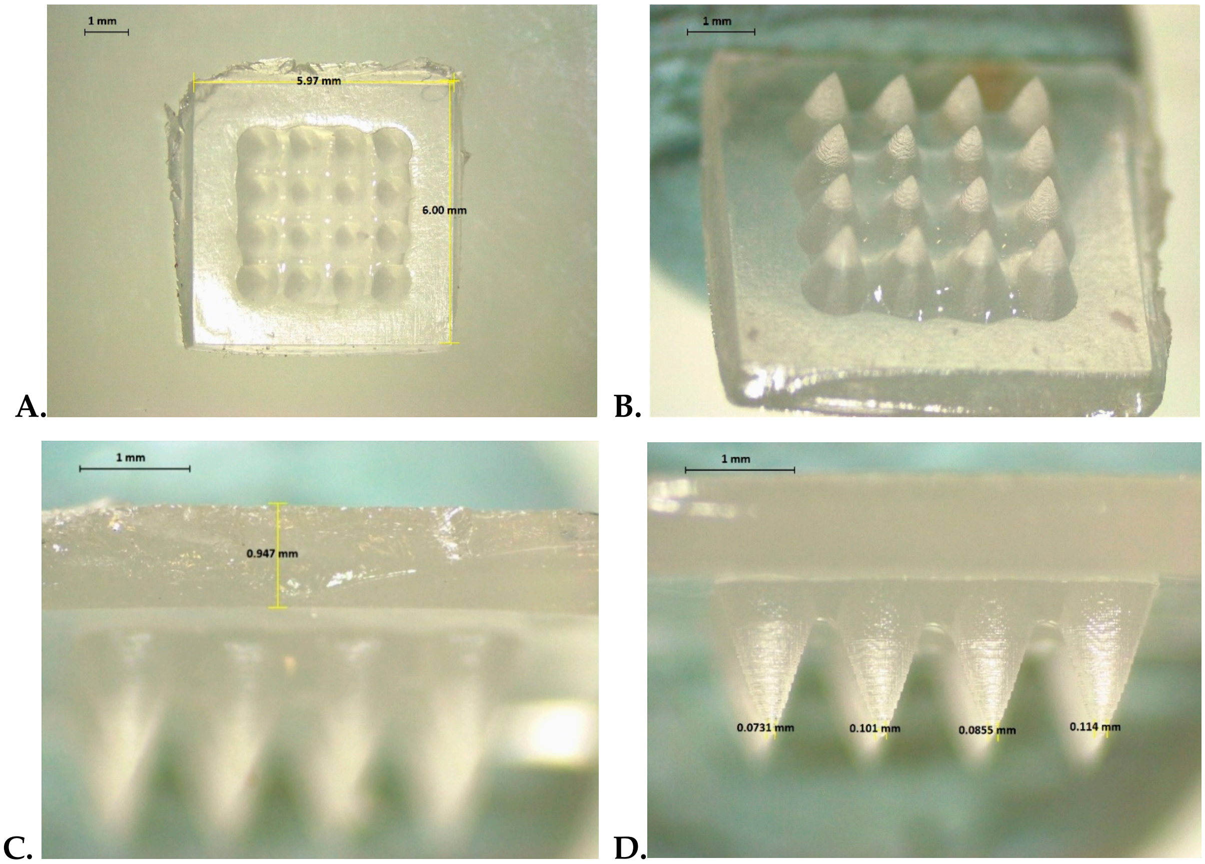

A. B. C. D.

Figure S1: Images of DLP-MN arrays fabricated using the optimised PEGDA/VP (40:60 w/w) resin formulation. (A) Top-view image showing overall patch dimensions (length ≈ 5.97 mm and width ≈ 6.00 mm) at 16× magnification. (B) Oblique-view image of the MN patch illustrating conical needle arrangement and uniform surface finish at 20× magnification. (C) Transverse-section view depicting the total patch thickness (≈ 0.95 mm) at 20× magnification. (D) Close-up view of individual MN tips, indicating measured tip diameters ranging from 0.07 to 0.11 mm at 25× magnification.

pharmaceutics

MDPI

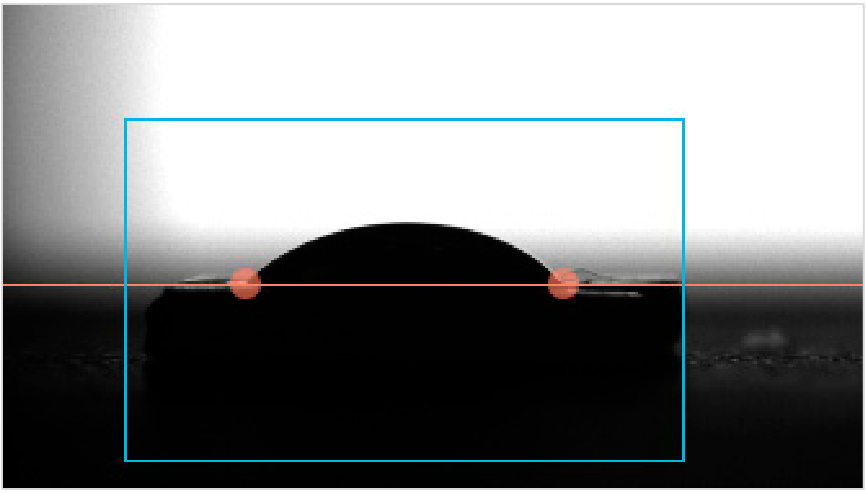

Figure S2: Static contact angle measurement of the PEGDA/VP microneedle patch using the sessile-drop method.
